# Choroid plexus epithelial monolayers – a cell culture model from porcine brain

**DOI:** 10.1186/1743-8454-3-13

**Published:** 2006-12-21

**Authors:** Carsten Baehr, Valeska Reichel, Gert Fricker

**Affiliations:** 1 Ruprecht-Karls-University, Institute of Pharmacy and Molecular Biotechnology, 69120 Heidelberg, Germany

## Abstract

**Background:**

The goal of the present study was to develop an *in vitro *choroid plexus (CP) epithelial cell culture model for studying transport of protein-mediated drug secretion from blood to cerebrospinal fluid (CSF) and *vice versa*.

**Methods:**

Cells were isolated by mechanical and enzymatic treatment of freshly isolated porcine plexus tissue. Epithelial cell monolayers were grown and CSF secretion and transepithelial resistance were determined. The expression of f-actin as well as the choroid plexus marker protein transthyretin (TTR), were assessed. The expression of the export proteins p-glycoprotein (Pgp, Abcb1) and multidrug resistance protein 1 (Mrp1, Abcc1) was studied by RT-PCR, Western-blot and immunofluorescence techniques and their functional activity was assessed by transport and uptake experiments.

**Results:**

Choroid plexus epithelial cells were isolated in high purity and grown to form confluent monolayers. Filter-grown monolayers displayed transendothelial resistance (TEER) values in the range of 100 to 150 Ωcm^2^. Morphologically, the cells showed the typical net work of f-actin and expressed TTR at a high rate. The cultured cells were able to secrete CSF at a rate of 48.2 ± 4.6 μl/cm^2^/h over 2–3 hours. The ABC-export protein Mrp1 was expressed in the basolateral (blood-facing) membranes of cell monolayers and intact tissue. P-glycoprotein showed only low expression within the apical (CSF directed) membrane but was located more in sub-apical cell compartments. This finding was paralleled by the lack of directed excretion of p-glycoprotein substrates, verapamil and rhodamine 123.

**Conclusion:**

It was demonstrated that CP epithelium can be isolated and cultured, with cells growing into intact monolayers, fully differentiating and with properties resembling the tissue *in vivo*. Thus, the established primary porcine CP model, allowing investigation of complex transport processes, can be used as a reliable tool for analysis of xenobiotic transport across the blood-cerebrospinal fluid barrier (BCSFB).

## Background

The brain is **very **sensitive to changes in its surrounding environment and homeostasis is essential to maintain normal function. Separated from the **vascular system**, most water-soluble compounds are excluded for stability and protection. Furthermore, endogenous metabolites as well as xenobiotics are actively removed from the CNS. Anatomically, three structures separate brain and blood flow: The network of brain capillaries or blood-brain barrier (BBB), the choroid plexus (CP) or blood-CSF barrier (BCSFB) and the arachnoid membrane. Both, the BBB and the BCSFB actively regulate type and concentration of molecules transported to and from the brain extracellular fluid, CSF and intracellular fluid.

The CP bears a resemblance to the renal proximal tubules in its epithelial ultrastructure and, like the kidney, transports near isotonic fluid across its epithelium [1]. Analogous to the kidney which functions to stabilize the chemical composition of blood, the CP is responsible for stability of the CSF. However, instead of acting as a filter, CSF is actively produced within the CP and enriched with nutrients derived from the blood. Bulk movement of fluid and molecules takes place from blood to CSF, across the infolded basolateral membrane and tightly packed villi at the apical membrane. However, movement may also occur in the opposite direction, with apical villi acting as a filter by secreting compounds and metabolites into the vascular system for eventual elimination by liver or kidney.

One of the most important CP functions is CSF secretion [2]. In man, its total volume is renewed every 4 to 5 h, with 90% of CSF produced by CP tissue [3]. The CP, with its array of metabolizing enzymes and coupled transepithelial vectorial efflux of conjugated metabolites into the blood, is an effective detoxification system within the brain [4]. In fact, the CP is one of the main sites of xenobiotic metabolism in the brain [5].

Many compounds are transported across the CP epithelium and to date, a total of eleven transporter families with almost 30 individual transport proteins, of which 13 are expressed at moderate to high levels, are found in the CP [6]. However, many are expressed at low levels and only eight are localized to specific membranes. The transporters are members of the solute carrier family (SLC) and of the active and energy consuming ATP-binding cassette (ABC) transporter family.

Several *in vivo *and *in vitro *techniques and models have been used to study transport across the CP [7]. *In vivo *techniques require elaborate experimental procedures and surgical skills. Common methods include the serial sampling of CSF, following drug administration, and deconvolution of data to determine transport profiles. Isolated tissue is used in extracorporal perfusion studies or for *in situ *chamber isolations of CP. *In vitro *methods include primary culture of isolated choroid plexus epithelial cells (CPEC), from a variety of different species, or culture of immortalized epithelial cells. Both primary and cell line CPEC cultures develop an impermeable monolayer and display the characteristics of CPEC *in vivo*.

Different approaches have been developed to establish a CPEC *in vitro *model. Crook *et al *in 1981 were the first to publish a protocol on epithelial cell isolation and cultivation using CP cells from bovine brains [8]. Other species used thereafter include rabbit [9,10], rat [11-13] and pig [14]. Typically, CP tissue is removed from lateral ventricles and mechanically fractionated. Individual CPs are digested using proteolytic enzymes, such as trypsin, pronase, collagenase and also DNAse, yielding an isolate of epithelial cells.

Here, we present a comprehensive functional characterization of an *in vitro *cell culture model, derived from porcine CP epithelial cells. In addition to investigation of features, such as marker protein expression, CSF secretion and enzyme function, special attention is directed towards the ABC transport proteins p-glycoprotein (Pgp) and multifunctional resistance protein 1 (Mrp1).

## Methods

### Tissue

Freshly isolated porcine brains were obtained from the local slaughterhouse (Fleischversorgungszentrum Mannheim, FRG).

### Isolation and culture of porcine CP epithelial cells

Epithelial cells from porcine CP were prepared as described previously with modifications [14]: Freshly isolated CP tissue was incubated with 0.25% (w/v) trypsin solution (Biochrom, Berlin, FRG) for 2.5 h at 4°C and then for an additional 0.5 h at 37°C. Proteolytic activity was terminated by addition of newborn calf serum (Biochrom, Berlin FRG). Remaining tissue was removed and the cell suspension was centrifuged at 1000 × g for 10 min. The cell pellet was resuspended in DMEM/HAM's F12 (1:1) (Bioconcept, Freiburg, FRG) supplemented with 10% (v/v) fetal calf serum (FCS), 4 mM L-glutamine, 5 μg/ml insulin, 200 ng/ml hydrocortisone, 5 ng/ml sodium selenite, 20 μM cytosine arabinoside, 100 g/ml penicillin/streptomycin and 10 ng/ml epidermal growth factor. Cells were seeded either on laminin-coated (50 μg/ml in water) Transwell^® ^filter membranes (Costar, Cambridge MA, USA) or flasks. The seeding density was adjusted that 1 g of tissue covered an area of 50 cm^2^. Twenty-four hours after plating, the cultures were washed with PBS (1 mM Ca^2+^, 0.5 mM Mg^2+^) to remove red blood cells. The medium was changed three times a week. FCS was removed after 9 days in culture (DIC).

### Total RNA isolation

Fresh tissue samples were stored in ice cold RNALater^® ^(Sigma, Steinheim, FRG) for transport in the time frame allowed by RNALater^® ^(max. 1 h) and frozen at -70°C upon arrival. Cultured CPEC were washed in KRB, pH 7.4, at 37°C three times to remove all remaining medium, before starting RNA isolation procedures. Total RNA from fresh and cultured CPEC was isolated according to the Qiagen RNeasy^® ^(Qiagen, Mannheim, FRG) protocol. Tissues or cells cultured in flasks were lysed in β-mercaptoethanol containing RLT buffer according to the Qiagen tissue and cell lysis protocols. An equal volume of 70% EtOH was added and samples were transferred to supplied spin columns. Following the Qiagen wash and spin regimen, total RNA was eluted in a small volume of nuclease free water. The amount of isolated RNA was quantified photometrically, measuring OD at 260 nm, with OD of 1 corresponding to 40 μg of single stranded RNA [15]. Purity was further established by taking the OD ratio of 260 nm over 280 nm. Only samples with a ratio of 1.7 to 2.0 were used, as low ratios are characteristic of protein and high ratios of residual β-mercaptoethanol contamination.

### Isolation and culture of porcine brain capillary endothelial cells

Brain capillary endothelial cells were isolated and cultured and permeability experiments were performed as described in our previous publication [16].

### Reverse Transcription

Reverse transcription (RT) was carried out according to the Promega Reverse Transcription System^® ^(Promega, Mannheim, FRG). One μg of total RNA was incubated at 70°C for 10 min and after adding the reaction mixture (in 20 μl: 4 μl 25 mM MgCl_2_, 4 μl 5X transcription buffer, 2 μl 10 mM dNTP mixture, 0.5 μl RNAsin^® ^ribonuclease inhibitor, 0.5 μg oligo(dT)15 primer and nuclease free water) containing 5 units of avian myeloblastosis virus reverse transcriptase (AMV-RT), was further incubated at 42°C for 60 min. Resulting cDNA was either used directly in further reactions or frozen at -20°C.

### Polymerase Chain Reaction (PCR)

The PCR reaction was used to amplify selective regions of generated cDNA. For each PCR reaction, 10 to 100% of the cDNA generated from 1 μg total RNA was used. All reactions were carried out using a hot-start protocol [17] in order to maximize sensitivity and specificity. The samples were run for a total of 15 to 50 cycles. Finally, PCR products were separated in a 1.5% ethidium bromide stained agarose gel and visualized under UV-light. All primers used are listed in Table 1.

**Table 1:** Primer Nucleotide Sequences.

Target*	*Accession Number*	*Forward Primer Reverse Primer*	*Product (bp)*
transthyretin (TTR, prealbumin)	X87846	TGGTCAAAGTCCTGGATGCT TTACATGCAAGCCTGTCCCT	371
β-actin	U07786	TTTGAGACCTTCAACACGCC TGATCCACATCTGCTGGAAG	703
Mrp1	L05628	TTCGTTCTCAGGCACATCAATG AACACAAGGATCTTCGTCTTCCTC	436
Pgp	AF016535	GAGAGGGGCCCAGTTGAGTG CACAAGCCCAAGACAGAAAGC	468

*Derived from porcine sequences if possible, if not human sequences (Mrp1, Pgp) were used.

### Semi-Quantitative RT-PCR

β-Actin expression was used as internal standard. Total RNA was isolated and transcribed, and selective regions amplified as described above. Density readings of ethidium bromide-stained PCR products were obtained, using the Scion Image software (NIH, USA), and normalized in relation to β-actin. To determine the phase of linear amplification, a series of amplifications in five-cycle intervals was run. A range of concentrations was amplified to ensure that results were not affected by initial amounts of mRNA and cDNA in any particular sample.

### Protein determinations

Total protein concentration was determined according to Bradford [18].

### Enzyme activities

Akaline phosphatase and γ-glutamyl transferase activities were measured according to the Sigma (Munich, FRG) protocol and to Caspers and Diglio [19]. Both enzymes were used as marker enzymes for the apical plasma membranes of CP epithelial cells.

### Immunocytological staining

CPEC were fixed in 95% EtOH for 15 min at room temperature. Cells were permeated with 0.1% Triton X-100 for 15 min. Specimens were then blocked in blocking buffer (1% BSA and 1% milk powder) for 1 h, before incubation with target antibody at a dilution of 1:10 in blocking buffer at room temperature overnight. After washing, samples were incubated with a FITC-labeled secondary IgG (Dako, Glostrup, DK) for 2 h at 1:100 in blocking buffer, with 10 μM propidium iodide (PI) added. For controls, the primary antibody was omitted. F-actin-stained cultured CPEC were incubated with 10 μM phalloidin-FITC and 10 μM PI or with PI only for treatment and control, respectively. Samples were embedded in Aqua Polymount^® ^(Polysciences Inc., Warrington, PA, USA) and viewed through an inverted Leica DM-IRB confocal microscope, using a 40× oil immersion objective (NA 1.2), a 488-nm argon ion laser excitation and a 500-nm long-pass emission filter.

### Isolation of apical and basolateral membrane fractions

Methods for CPEC membrane fraction isolation were adapted from a protocol for kidney membranes [20]. Freshly isolated lateral CP was homogenized in homogenizing solution (HS, in mM: 300 mannitol, 0.1 phenylmethylsulfonyl fluoride, 1 EDTA and 18 Tris HCl, pH 7.5), using 6 g CP tissue per 50 ml HS. The homogenate was centrifuged at 3,000 g_(max) _for 10 min. MgCl_2_(1 M at 1 % of volume) was added to the resulting supernatant (S1) and stirred for 20 min on ice. The solution was then centrifuged at 2,000 g_(max) _for 10 min. The resulting supernatant (S2) was used for isolation of apical membrane fractions and resulting pellet (P2) for the isolation of basolateral membrane fractions.

For the isolation of apical membrane fractions, S2 was centrifuged at 33,000 g_(max) _for 20 min. The pellet was resuspended in half the original volume of HS, and 1 M MgCl_2 _was added to arrive at a concentration of 10 mM MgCl_2_. After 20 min on ice, the solution was centrifuged at 4,350 g_(max) _for 12 min and the pellet discarded. This procedure was repeated twice. Finally, the solution was centrifuged at 6400 g_(max) _for 12 min, the pellet discarded and the supernatant further centrifuged at 32,900 g_(max) _for 20 min. The resulting pellet was the apical membrane fraction. Basolateral membrane fractions were obtained by resuspending P2 in HS and centrifuging at 48,300 g_(max) _for 20 min. This procedure was repeated twice. Then the pellet was resuspended in a small volume of HS and mixed with 2 ml Percoll per 10 ml. The mixture was centrifuged 48,300 g_(max) _for 30 min, after which two layers became evident. The upper layer was removed and remaining Percoll washed out by adding an excess amount of PBS (without magnesium and calcium) and centrifuging at 48,300 g_(max) _for 30 min. The pellet was taken up in HS. Both membrane fractions were characterized, the apical by analysis of alkaline phosphatase and γ-glutamyl transferase activity, the basolateral by determination of Mrp1 expression in Western blot analysis.

### Western Blots

CPEC whole cell protein samples or enriched membrane fractions were studied by Western blot analysis according to Sambrook *et al *[15]. Proteins were denatured in a SDS-containing sample buffer (100 mM Tris-HCl (pH 6.8), 4% SDS, 2% bromophenol blue, 20% glycerol) and reduced using either β-mercaptoethanol or D,L-dithiothreitol. Protein samples were heated to 95°C or 60°C for 10 min or 5 min for analysis of marker proteins or ABC-transporters, respectively. Following SDS-PAGE (15 to 6% polyacrylamide gels) at 80 V for 2 h in running buffer (25 mM tris (pH 8.3), 250 mM glycine, 0.1% SDS), samples were blotted on nitrocellulose at 250 mA for 2.5 h in blotting buffer (39 mM glycine, 48 mM tris-base (pH 8.3), 0.037% SDS, 20% methanol). After 1 h blocking, samples were incubated with target antibody at a dilution of 1:100 in blocking buffer at 4°C overnight, followed by incubation in horseradish peroxidase (HRP)-labeled secondary IgG (DakoCytomation) at a dilution of 1:1,000 in blocking buffer for 2 h. The resulting bands were visualized with diaminobenzidine in combination with NiCl_2_. Porcine brain capillary endothelial cells, liver and kidney samples were used as positive controls.

### CPEC Cerebrospinal-Fluid Secretion

To assess CSF secretion by cultured CPEC, cells were cultured on permeable, polyester six-well Transwell^® ^filter plates coated with 5 μg/ml laminin for 13 to 15 DIC. Cells were washed three times in CSF secretion buffer (CSFB, in mM: 122 NaCl, 4 KCl, 1 CaCl_2_, 1 MgCl_2_, 15 Na-HCO_3_, 15 HEPES, 0.5 Na_2_HPO_4_, 0.5 NaH_2_PO_4 _and 17.5 glucose, pH 7.3, 37°C, with 5 μg/ml insulin added [21]). Following a 1 h preincubation with CSFB, 1.0 ml 1.0 μM 67 kDa fluorescent-dextran (FITC-dextran) in CSFB was added to both apical and basolateral chambers and secreted CSF volume (in μl/cm^2^) was calculated as a function of FITC-dextran concentration change. Dextran at 67 kDa is suitable for these measurements, as it was shown in separate experiments to be impermeable to the cell monolayer.

Filters were incubated at 37°C, 5% CO_2 _and 95% relative humidity for up to 8 h. 200 μl samples were taken on the hour from either the apical or basolateral chamber and replaced with fresh dextran solution. Samples were analyzed in a fluorescent plate reader (Ascent Fluoroscan, Oy, Finland). CSF volumes secreted were corrected for the sampling regime (for details on correction for sampling regime see Hakvoort *et al *[21]) and then calculated according to equation (1):

VCSF=CDex (initial)−CDex (final)CDex (final)×V0     (1)
 MathType@MTEF@5@5@+=feaafiart1ev1aaatCvAUfKttLearuWrP9MDH5MBPbIqV92AaeXatLxBI9gBaebbnrfifHhDYfgasaacH8akY=wiFfYdH8Gipec8Eeeu0xXdbba9frFj0=OqFfea0dXdd9vqai=hGuQ8kuc9pgc9s8qqaq=dirpe0xb9q8qiLsFr0=vr0=vr0dc8meaabaqaciaacaGaaeqabaqabeGadaaakeaacqqGwbGvdaWgaaWcbaGaee4qamKaee4uamLaeeOrayeabeaakiabg2da9maalaaabaGaee4qam0aaSbaaSqaaiabbseaejabbwgaLjabbIha4jabbccaGiabcIcaOiabbMgaPjabb6gaUjabbMgaPjabbsha0jabbMgaPjabbggaHjabbYgaSjabcMcaPaqabaGccqGHsislcqqGdbWqdaWgaaWcbaGaeeiraqKaeeyzauMaeeiEaGNaeeiiaaIaeiikaGIaeeOzayMaeeyAaKMaeeOBa4MaeeyyaeMaeeiBaWMaeiykaKcabeaaaOqaaiabboeadnaaBaaaleaacqqGebarcqqGLbqzcqqG4baEcqqGGaaicqGGOaakcqqGMbGzcqqGPbqAcqqGUbGBcqqGHbqycqqGSbaBcqGGPaqkaeqaaaaakiabgEna0kabdAfawnaaBaaaleaacqaIWaamaeqaaOGaaCzcaiaaxMaadaqadaqaaiabigdaXaGaayjkaiaawMcaaaaa@693E@

Where, V_CSF _= CSF volume [μl], CDex (initial) = initial FITC-dextran concentration [μM], CDex (final) = final FITC-dextran concentration [μM], V_0 _= initial volume applied [μl].

### Transepithelial Electrical Resistance (TEER)

TEER values were determined for CPEC grown on permeable polyester Costar Transwell^® ^membranes, using the Millicell^®^-ERS and STX-2 electrode system (World Precision Instruments, Berlin, FRG). Control values obtained from coated filters without cells (blank) were subtracted and the resulting values multiplied by the filter surface area, resulting in TEER values of Ωcm^2^.

### Permeability experiments

For paracellular permeability analysis, CPEC were grown on Transwell^® ^filter systems for 9 DIC in serum-containing medium, followed by another 5 DIC in serum-free medium. Marker compounds were applied from either the apical or basolateral chamber and accumulation was measured in the opposite compartment. All markers were used at a concentration of 10 μM. Only filter systems showing a TEER higher than 100 Ωcm^2 ^(≈ 600 Ωcm^2 ^following impedance analysis [22]) were used for permeability experiments. The apparent permeability coefficients were measured for 5-carboxyfluorescein (0.4 kDa) and fluorescent-labeled dextrans between 4.4 kDa and 500 kDa in size, across CPEC 14 DIC monolayers, grown on polyester membranes of 10 μM thickness, a pore size of 0.4 μm, and 4 × 10^6 ^pores per cm growth area. The medium was exchanged with Krebs-Ringer buffer (KRB), 0.5 ml in the apical, 1.5 ml in the basolateral chamber, and test compound added to the donor compartment at the desired concentration. The plates were shaken at 100 rpm and 100 μl samples were taken after 15, 30, 45, and 60 min from the acceptor compartment and replaced with fresh buffer. For inhibitor treatments, cells were pre-incubated with inhibitor alone. For pre-incubation and for transport measurements, inhibitors were added to both apical and basolateral compartments.

The permeability coefficient *P *of test compounds was calculated according to equation (2):

P=dcdt×VacceptorA×c0     (2)
 MathType@MTEF@5@5@+=feaafiart1ev1aaatCvAUfKttLearuWrP9MDH5MBPbIqV92AaeXatLxBI9gBaebbnrfifHhDYfgasaacH8akY=wiFfYdH8Gipec8Eeeu0xXdbba9frFj0=OqFfea0dXdd9vqai=hGuQ8kuc9pgc9s8qqaq=dirpe0xb9q8qiLsFr0=vr0=vr0dc8meaabaqaciaacaGaaeqabaqabeGadaaakeaacqWGqbaucqGH9aqpdaWcaaqaaiabdsgaKjabdogaJbqaaiabdsgaKjabdsha0baacqGHxdaTdaWcaaqaaiabdAfawnaaBaaaleaacqWGHbqycqWGJbWycqWGJbWycqWGLbqzcqWGWbaCcqWG0baDcqWGVbWBcqWGYbGCaeqaaaGcbaGaemyqaeKaey41aqRaem4yam2aaSbaaSqaaiabicdaWaqabaaaaOGaaCzcaiaaxMaadaqadaqaaiabikdaYaGaayjkaiaawMcaaaaa@4C1D@

with *dc/dt *representing the change of the acceptor concentration over time, V_*acceptor *_the acceptor volume, *A *the membrane surface area and *c*_*0 *_the initial donor concentration. Concentrations were assessed by analysis in a fluorescent plate reader (Ascent Fluoroscan) for fluorescent-labelled compounds. When verapamil was used, tracer amounts of [^3^H]-verapamil (NEN/Perkin-Elmer, Rodgau – Jügesheim, FRG) were added, and the samples were analyzed by liquid scintillation counting.

### CP Uptake assays

Uptake assays were carried out with CPEC cultured in 96-well plates. For all experiments CPEC were cultured for 13 DIC up to 15 DIC. Cells were washed three times and then pre-incubated in KRB, pH 7.4, 37°C, for 30 min, without (control) and with inhibitor added. After 90 min incubation with substrate or substrate and inhibitor for control and treatment samples, respectively, cells were washed five times with KRB, pH 7.4, 37°C, lysed in 1% Triton X-100 and the wells analyzed in a fluorescent plate reader (Ascent Fluoroscan). Wells containing CPEC incubated with KRB only served as blank. Apparent uptake was calculated by subtracting blanks from fluorescent values. Fluorescent values were quantified using an appropriate standard curve.

### Statistics

All values presented are means ± SEM. Control and treatment groups were compared by either student's t-test or one-way analysis of variance, followed by either a Bonferroni or Dunnett's post hoc test. Differences were considered significant at *P < 0.05 and **P < 0.01. Regression analysis was carried out to analyze appropriateness of standard curves used and only linear relationships with an R^2^-value greater than 0.95 were used for transformations.

## Results

### Cell isolation and culture

Porcine lateral CP isolated from brain and digested by cold trypsination yielded 6.5 × 10^6 ^± 5.2 × 10^5 ^viable cells/g tissue (n = 25), using approximately 4.2 g tissue per preparation. Following isolation, cells were seeded on either Transwell^® ^filter systems, 96-well plates or in culture flasks at a seeding density of at least 1 g per 50 cm^2 ^or 1.2 × 10^5 ^cells per cm^2^. In general, CPEC growth was slow and proliferation rates were low. Addition of cytosine arabinoside suppressed the growth of contaminating cells and epithelial cell cultures developed into a monolayer within nine DIC, displaying the typical cobblestone appearance. After nine DIC, serum was removed from the medium and CPEC monolayers allowed to fully differentiate for another 4–5 days.

### CP epithelial cell F-actin

**Figure 1:**
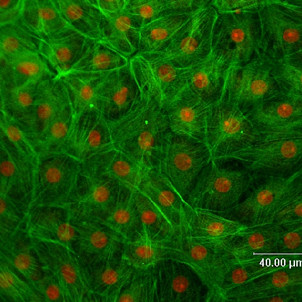
FITC-Phalloidin-stained porcine choroid plexus epithelial cells monolayer after 14 days cell culture (stained f-actin in green, propidium iodide-stained cell nuclei in red).

Actin is most abundant in eukaryotic cells and highly conserved. Actin filaments are associated with tight junctions (TJ) and perijunctional actin is directly involved in controlling paracellular permeability [23]. To visualize actin distribution throughout the cultured cells, the protein was stained with fluorescent-labeled phalloidin (FITC-phalloidin), which binds specifically to f-actin. Propidium iodide (PI) was also added to permeated cells, staining cell nuclei. Figure 1 shows an image of CPEC 14 DIC after incubation with FITC-phalloidin and PI, visualized by confocal laser scanning microscopy. Overall, cultured epithelial cells appeared as a confluent monolayer. No undesired and contaminating cells (e.g. fibroblasts or macrophages) were present. All CPEC abundantly expressed f-actin and, as expected from fully differentiated cells, the stain revealed actin filaments in networks and bundles, some of which spanned the whole cell.

### Marker protein prealbumin (transthyretin, TTR)

**Figure 2:**
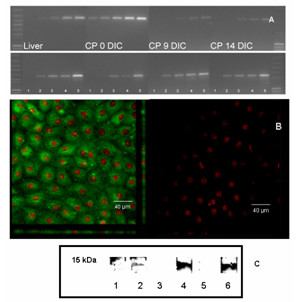
A) Amplified cDNA of liver (control), fresh CP, CP 9 DIC, and 14 DIC in an ethidium bromide gel. TTR amplifies as 371 bp (top row), β-actin, used as internal standard amplifies at 703 bp (bottom row). For each sample, lanes 1 through 5 show amplification results for 15 to 35 cycles, with lanes corresponding to PCR products with 5 cycles of additional amplification (lane 1, 15; lanne 2,20, lane 3,25; lanne 4,30; lane 5, 35). B) Confocal images of choroid plexus epithelial cell monolayer 14 DIC. On the left, TTR is shown in green after incubation of the cells with anti-TTR and secondary antibody. Cell nuclei are shown in red (propidium iodide staining). No TTR staining was seen in control images (right side), when cells were incubated with secondary antibody only. C) TTR Western blot of liver (lanes 1 and 2), freshly isolated CP (lanes 3 and 4) and choroid plexus epithelial cells 14 DIC (lanes 5 and 6). Samples without primary antibody served as controls (lanes 1, 3 and 5).

In the mammalian brain, TTR is specifically localized to the CP [24] and hence TTR is a convenient marker for CPEC. The unpublished mRNA sequence for porcine specific TTR, accession number X87846, was submitted to the Nucleotide Entrez database by Archibald *et al *[25]. For TTR mRNA expression analysis, cells were grown for nine DIC in serum-containing medium and allowed to fully differentiate in serum-free medium for additional five days. Figure 2A shows amplified cDNA of porcine liver (control), fresh CP, CP 9 DIC and CP 14 DIC in an ethidium bromide gel visualized under UV-light. TTR amplified at 371 bp specific for TTR mRNA expression and is shown in the top lanes. Fresh CP (0 DIC) TTR amplification produced gel bands after 15 amplification cycles. The signal for cultured CPEC was less intense, with signals visible after 25 amplification cycles. β-Actin amplified with similar intensity in all CPEC samples. Thus, TTR mRNA expression was higher in fresh CP tissue when compared to TTR expressed in cultured CPEC.

TTR protein expression was investigated in immunological staining of cultured CPEC 14 DIC and in Western blot analysis, using fresh and cultured CPEC 14 DIC. Figure 2B shows confocal images of a cultured CPEC monolayer stained with anti-TTR and PI. In cultured CPEC, TTR localized throughout the cytoplasm, with most intense staining around the cell nuclei. Fewer signals were observed towards the cell membrane.

Figure 2C shows the Western blot of the 55 kDa TTR denatured to 15 kDa TTR fragments (monomer subunits) in fresh CPEC and cultured cells 14 DIC. Porcine liver was used as positive control. Samples without primary antibody incubation are included as negative control. All samples with primary antibody added show TTR staining.

### Enzyme analysis

**Figure 3:**
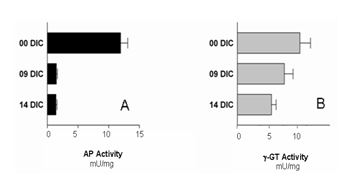
Alkaline phosphatase (A) and γ-glutamyl-transferase (B) activity in freshly isolated choroid plexus epithelial cells and cells 9 DIC and 14 DIC (means ± SEM, n = 6).

Two enzymes, alkaline phosphatase (AP; EC 3.1.3.1) and γ-glutamyl transferase (γ-GT; EC 2.3.2.1), were used to characterize cultured CPEC. Both membrane-bound enzymes are present at the BCSFB in different species, including pig, and have been previously used to characterize CP membrane preparations [26,27]. Activities were determined for cultured CPEC at nine DIC, before serum was removed from culture medium, and at 14 DIC, after cells were fully differentiated. Measured enzyme activity was compared to freshly isolated CPEC (0 DIC). AP activity (Figure 3A) decreased from 11.9 mU/mg down to 1.5 mU/mg and 1.4 mU/mg from 0 DIC to 9 DIC and 14 DIC, resembling an 87.4% and 88.2% decrease compared to activity measured after cell isolation. The reduction in γ-GT (Figure 3B) was more gradual, with values decreasing from 11.4 mU/mg to 9.5 mU/mg and 5.0 mU/mg from 0 DIC to 9 and 14 DIC, resembling a 16.7% and 56.1% decrease after 14 DIC.

### Cultured epithelial cell Cerebrospinal-Fluid Secretion

The high rate of CSF secretion *in vivo *is fuelled by the rapid blood flow through the vascular supply to the plexus (5 ml/g/min), which is about tenfold higher than average cerebral blood flow [28]. *In vitro*, CSF secretion was measured from cells grown on 6-well Transwell^® ^filter systems. FITC-dextran was added to the CSFB and secreted CSF volume (in μl/cm^2^) calculated as a function of FITC-dextran concentration change.

**Figure 4:**
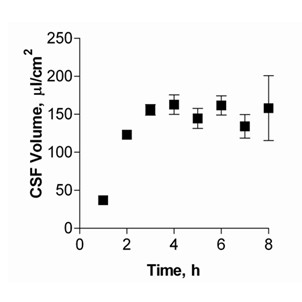
Choroid plexus epithelial cell secretion volumes after 14 DIC. Measurements were taken every hour up to 8 h (means ± SEM, n = 6).

Figure 4 shows the CSF volume secreted hourly by cultured CPEC for up to 8 h. The volume of CSF secreted by the monolayer increased in linear fashion, reaching a plateau after 5 h of approximately 150 μl/cm^2^. No further increase in CSF volume secreted per time interval was seen thereafter. Using measurements made during the first 4 h of linear CSF secretion, CSF production rates were measured to be 48.2 ± 4.6 μl/cm^2^/h. The volume of CSF produced in this model system is similar to results obtained in previous studies [21], when a secretion rate of approximately 45 μl/cm^2^/h was measured over 4 h of incubation using a slightly different calculation. In another study, saturation of CSF production was reached after 6 h, with 12% of the basolateral chamber volume secreted into the apical compartment [29]. In our studies, saturation was reached after 5 h with an increase of approximately 15% in apical volume. A possible reason for fluid secretion reaching a plateau may be due to limitations imposed by the culture system if there is an increase in proton concentration in the basolateral chamber over time, which inhibits Na^+^/H^+ ^exchange and thus fluid secretion (since the basolateral fluid was not replaced) as described by Hakvoort *et al*. [21].

### Cultured CP Monolayer Transepithelial Resistance (TEER) Values

TEER values have been used extensively as a measure for leakiness of cellular barriers. In the present study, CPEC monolayers displayed TEER values in the range of 100 to 150 Ωcm^2 ^and were judged confluent at values > 100 Ωcm^2^. Due to the anatomical location of the CP, there is no data on TEER *in vivo *available for mammals and no TEER information on lateral CP TEER for any species. Fourth ventricle CP TEER measurements were carried out in the bullfrog with values at approximately 170 Ωcm^2 ^[30]. Elasmobranch resistance values were measured with values of 70 to 107 Ωcm^2 ^[31]. In primary cultures of rat CPEC, TEER values were in the range of 100–500 Ωcm^2 ^[11,12]. Porcine CPEC models displayed resistance values in the range of 100 to 170 Ωcm^2 ^[14,21]. One group reported TEER values exceeding 1500 Ωcm^2^, for cells grown in serum-free medium, with higher values resulting from the specific method of impedance analysis [22,32]. However, these resistance measurements were taken with a different device to that used here. The values obtained in the present study are equivalent to values of > 600 when quantified by impedance analysis.

### Permeability marker analysis

**Figure 5:**
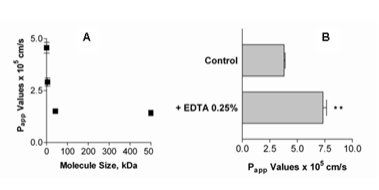
A) Apparent permeability coefficients of 5-carboxyfluorescein and dextrans 0.4 kDa to 500 kDa (means ± SEM, n = 6). B) Permeability of 5-carboxyfluorescein without and with open tight junctions by the addition of EDTA (means ± SEM, n = 6).

For cells of the BCSFB *in vitro*, the experimental conditions influence cell differentiation, including tight junction (TJ) expression. Even though *in vitro *models can be a good tool for assessing epithelial transport, measurements tend to overestimate *in vivo *drug movement and correlation between both types of data is necessary. A wide range of compounds has been used to characterize BBB and BCSFB membranes, including the zero-permeability marker, PEG-4000 (FDA, Biopharmaceutical Classification System: Guidance for Industry, 2000). In these experiments, P_app _(apparent permeability coefficient) values ranged from 4.56 ± 0.26 × 10^-5 ^cm/s for smallest down to 1.42 ± 0.13 × 10^-5 ^cm/s for largest molecules (Figure 5A). Notably, permeability decreased to a plateau at approximately 40 kDa, with P_app _values for 42 kDa dextrans not significantly different from P_app _for 500 kDa dextrans.

Further evidence for CPEC TJ expression was gained from comparing permeability across monolayers with intact or opened TJs. One method of opening TJs is to remove free calcium from surrounding medium. The transport of 5-carboxyfluorescein (10 μM) in CPEC 14 DIC, was measured without and with 0.25% EDTA added to the medium. Permeability of the paracellular marker was significantly higher through monolayers with opened TJs, with P_app _values nearly doubling (Figure 5B).

### ABC-Transport protein gene expression

Active excretion by ABC efflux transporters not only removes endogenous metabolites and waste products from the brain, but also limits uptake and penetration of many therapeutic compounds [33]. Expression of ABC transporters has been extensively studied at the BBB. With delivery across the CP gaining importance and only limited information regarding ABC transporters at the BCSFB available, further investigation into the role of these transporters in CPEC is crucial. Two proteins contributing to multidrug resistance are of particular importance due to the large number and diversity of compounds transported: the MDR phenotype Pgp, part of the ABCB subfamily, and Mrp1, part of the ABCC subfamily. Compared to the BBB, relatively little is known about gene expression of ABC-transport proteins Pgp and Mrp1 in CP and data on CPEC Pgp and Mrp1 mRNA is only available for rat [6,34].

Pgp and Mrp1 gene expression at the BCSFB was analyzed on a qualitative and quantitative basis and the distribution and localization of both proteins to polarized CPEC membranes determined. For Pgp and Mrp1 gene expression analyses, porcine RNA was isolated from fresh CP, cultured CPEC 14 DIC and liver tissue (positive control). To account for sample-to-sample variation, β-actin amplification was run simultaneously.

**Figure 6:**
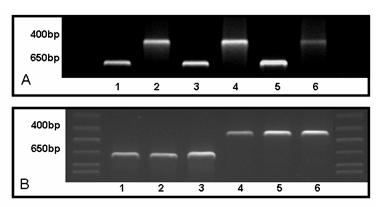
A) RT-PCR of Pgp (468 bp) and β-actin (703 bp); from left to right: liver (lanes 1 and 2), fresh CP tissue (lanes 3 and 4) and CPEC 14 DIC (lanes 5 and 6). B) RT-PCR of Mrp1 (436 bp) and β-actin (703 bp) in CPEC 14 DIC (lanes 1 and 4), fresh CP tissue (lanes 2 and 5) and liver (lanes 3 and 6).

Comparative RT-PCR experiments from CP tissue and cultured epithelial cell monolayers indicated the presence of Pgp, thus corroborating recent findings in intact rodent tissue [35]. Figure 6A shows the amplified 468 bp Pgp DNA product and β-actin product at 703 bp visualized under UV-light. Pgp was amplified for 50 cycles and β-actin for 35 cycles. Liver mRNA was used as positive control. Fresh and cultured CPEC expressed Pgp mRNA at low levels. Similar results, in terms of expression level, were obtained in previous studies [6]. However, it must be noted that the signal for Pgp was weaker in cultured epithelial cells as compared to fresh tissue (Figure 6A). The Pgp/β-actin density ratios amounted to 1.26 in liver tissue, which was used as control, compared to 1.14 at fresh Cp tissue, but only to 0.33 at cells at 14 DIC. The weaker signal in cultured epithelial cells is comparable to the reduction previously seen in freshly isolated and cultured microvessel endothelial cells [16].

Figure 6B shows the amplified 436 bp Mrp1 DNA product and β-actin product at 703 bp. Mrp1 was amplified for 50 cycles and β-actin (internal standard) for 35 cycles. Analysis revealed Mrp1 mRNA expression in fresh and cultured CPEC. Liver mRNA was again used as a positive control. For a semi-quantitative analysis, Mrp1 was amplified at 30 cycles and mRNA of cultured CPEC 14 DIC was compared to freshly isolated CPEC. After normalization to β-actin RNA, there was no significant difference between Mrp1 gene expression in cultured CPEC 14 DIC and freshly isolated CPEC (Figure 6B). The MRP1/β-actin density ratio in liver tissue was 1.00, compared to 1.19 for fresh CP tissue and 0.93 for cells 14 DIC.

**Figure 7:**
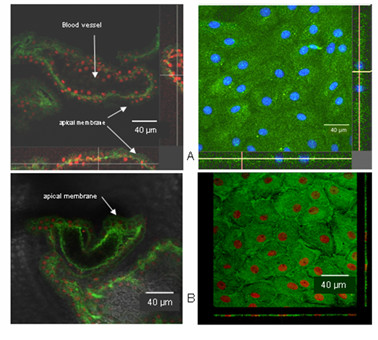
A) Confocal images of choroid plexus epithelial cell monolayers (right side) and freshly isolated intact tissue (left side). Pgp (immunostaining shown in green) is localized sub-apical (intracellular) rather than in apical membranes. B) Immunostaining of Mrp1 in 14 day-old cell monolayers (right side) and freshly isolated intact tissue (left side). Immunostaining (green) occurs predominantly in the abluminal (basolateral) side.

Immunostaining confirmed the differences in Pgp and Mrp1 expression: The monoclonal antibody C219 (Alexis, Grünberg, FRG) was used as primary antibody for Pgp immunofluorescence. The Pgp protein, stained green, showed a subapical and possible intracellular localization, rather than a clear membrane-associated distribution both in intact tissue and in cultured epithelial cells (Figure 7A). This suggests that localization of Pgp in CP may be different from that in other epithelial cells.

Mrp1 protein was also visualized in freshly isolated CP and cultured CPEC (Figure 7B). Epithelial cells were stained with MRPr1 antibody (Alexis, Grünberg, FRG), previously shown to bind specifically to its antigen [36]. The image depicts a large single CP blood vessel, surrounded by a single layer of epithelial cells. All epithelial cell nuclei within the focal plane are shown in red. The inside of CP blood vessels appears black. Mrp1 was clearly present in CP epithelium. Staining was most intense towards the basolateral, blood side of the epithelium. Thus, Mrp1 is expressed and localized to the basolateral membrane. Mrp1 expression was also analyzed in CPEC 14 DIC. Figure 7B, right side, shows one *xy*-section and a digitally reassembled construct of 20 *xy*-sections taken at 1 μm intervals. As in fresh CP tissue, Mrp1 is highly expressed in cultured CPEC, and is concentrated at the basolateral membrane.

**Figure 8:**
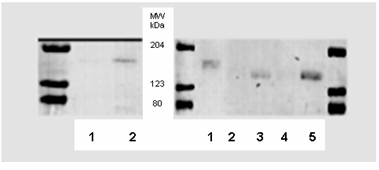
Western blot of Mrp1 (left side; primary antibody MRPR1) and Pgp (right side; primary antibody: Alexis C219). Left side: lane 1, apical CP membranes; lane 2, basolateral CP membranes; Right side: lane 1, brain capillary endothelial cells (20 μg); lane 2, basolateral choroid plexus epithelial cells membranes (10 μg); lane 3, apical membrane (10 μg); lane 4, basolateral membrane (25 μg); lane 5, apical membrane (25 μg).

To further clarify the localization of Pgp in epithelial cells, Western blots of Pgp with isolated membrane fractions were performed. Apical and basolateral fractions were isolated. Based on the activity of alkaline phosphatase and γ-glutamyl transferase, the enrichment over homogenate of apical (CSF oriented) membranes was 27.3 ± 0.8 fold and 27.7 ± 1.0, respectively. Western blot analysis showed that Mrp1 was localized exclusively in the basolateral (blood facing) membrane (Figure 8). For Pgp, Western blots of these membrane fractions excluded basolateral localization but a weak signal was found in apical membrane fractions (Figure 8).

### Functional studies with Pgp

Functional characterization of Pgp was carried out in CPEC 14 DIC using the Pgp substrates rhodamine 123 and verapamil. Uptake of various concentrations of rhodamine 123 into CPEC 14 DIC cultured in 96-well plates was measured and the effects of transport inhibitors with known affinity for Pgp assessed.

The mainly intracellular localization of Pgp leaves room for speculation as to if and how compounds are excluded from CPEC. Should Pgp be inserted into the apical membrane of the epithelium, Pgp substrates would be transported back into the medium. Concomitant incubation with inhibitors would reduce efflux and lead to an increase in cellular substrate uptake. If Pgp localized to cytoplasmic vesicles, substrate would be trapped within the cellular vesicles. Exocytosis of substrate-filled vesicles would reduce substrate concentrations. Effects of inhibitors on intracellular vesicularized Pgp would only alter cellular concentrations if basolateral exocytosis occurs, leading to an increase in cellular substrate concentrations.

**Figure 9:**
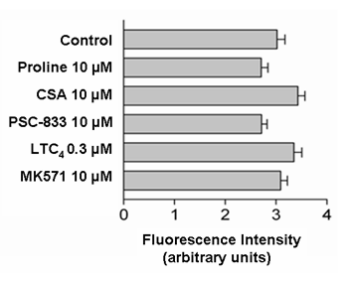
Uptake of 2 μM rhodamine 123 without (control) and with substrates of ABC-transport proteins. None of the compounds showed a significant effect on uptake of the Pgp substrate rhodamine 123 (CSA = cyclosporine A, PSC = PSC-833; LTC4 = leucotriene C_4_; means ± SEM, n = 6).

To functionally characterize Pgp activity, cells were incubated with 2 μM of rhodamine 123 for 1 hour with Pgp-substrates or without (control). Proline was used as a secondary control. In cultured CPEC none of the effectors, including cyclosporine-A (CSA) and its analog PSC883, caused a significant alteration in 2 μM rhodamine 123 uptake over 1 h (Figure 9), suggesting that Pgp is not functionally active in the membranes of this cell culture model.

In addition to studies of rhodamine 123, the transport of verapamil was measured in CP epithelial cells grown on permeable filter supports. Permeability was determined from apical to basolateral (a → b) and from the basolateral to the apical side (b → a) of cells. The transport of verapamil across brain capillary endothelial cells was also measured as a control. Contrary to capillary endothelial cells, where a preferential net transport from the abluminal (al) to the luminal (l) compartment was seen (ratio al → l/l → al: 8.27; in presence of 10 μM Pgp blocker PSC-833: 1.25), no preferred transport was observed in CP epithelial cells in either apical (a) or basolateral direction (b) (ratio a → b/b → a: 1.22). This result confirms the findings with rhodamine 123 and makes it likely, that Pgp is not functionally active in cultured CP cells (Table 2).

**Table 2:** Permeability of verapamil across isolated brain capillary endothelial cells and isolated choroid plexus epithelial cells kept in monolayer cultures.

		**mean permeability coefficient (× 10^-5^cm/sec)**	**ratio**
**Brain capillary endothelial cells**	luminal (l) → abluminal (al)	4.68	al → l/l → al (brain→blood/blood→brain) 8.27
	abluminal → luminal	38.70	
**CP epithelial cells**	apical (a) → basolateral (b)	11.65	a → b/b → a (CSF→blood/blood→CSF) 1.22
	basolateral → apical	9.55	

### Functional studies with Mrp1

**Figure 10:**
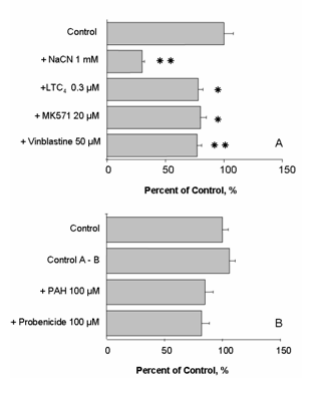
**A) **Transport of fluorescein-methotrexate across cultured choroid plexus epithelial cells **(14 DIC) **from the apical to the basolateral side in absence and presence of modifiers of organic anion transport expressed as a percentage of control (means ± SEM, n = 6). B) Transport of fluorescein-methotrexate across cultured choroid plexus epithelial cells from the basolateral to the apical side in absence and in presence of modifiers of organic anion transport. None of the compounds showed a significant effect on Fl-MTX permeability (means ± SEM, n = 6).

Secretion of the Mrp-substrate Fluorescein-Methotrexate was analyzed in cultured CPEC and fully differentiated at 14 DIC. Thereby, transport of FL-MTX across CPEC grown on Transwell^® ^filter systems was determined, allowing direct access to the blood-facing side of CPEC. The metabolic inhibitor NaCN reduced transport of 2 μM FL-MTX from the apical to the basolateral compartment by more than 50% (Figure 10a), as seen in *ex vivo *tissue [37]. Incubation with organic anions leukotriene C_4 _(LTC_4_), MK571 and vinblastine significantly reduced FL-MTX transport from apical to basolateral compartments.

Transport of 2 μM FL-MTX from basolateral to apical chambers was not significantly different from apical to basolateral secretion (Figure 10b). Incubation with other organic anions, including *p*-aminohippuric acid and probenecid did not significantly affect transport of FL-MTX. Hence, FL-MTX transport from the apical to basolateral chambers was affected by inhibitors but not *vice versa*. Regarding molecular and immunohistochemical data, inhibitors NaCN, LTC_4 _and MK571 are most likely targeting Mrp1, expressed at the basolateral membrane of CPEC. Thus, it is likely that transport of FL-MTX was reduced due to decreased efflux on Mrp1.

## Discussion

The goal of the present study was the development of a CP epithelial cell culture model, which allows drug transport from blood to CSF and *vice versa *to be studied *in vitro*. Morphological and functional characterization of such a model is an indispensable prerequisite to estimate its predictive character. It has been demonstrated that porcine CP epithelium can be isolated and cultured, with cells growing into intact monolayers, fully differentiating and with properties resembling the tissue *in vivo*. Monolayers were formed approximately at day 9 after plating, which was faster than described previously [38] with monolayer formation observed with rat CPECs at 12–14 days, but similar to measurements of monolayer formation with rat CPEC at day 8 [12]. In another study with CPECs from sheep [39], optical confluence of cultured cells appeared at days 2–3 after seeding, with TEER values significantly increased between days 4 and 6 and a steady state TEER value of 85 ± 9 Ωcm^2 ^reached at day 8. In our system TEER values of 100–150 Ωcm^2^were determined at confluence with an apparent permeability coefficient of 5-carboxyfluorescein of 4.5 × 10^-5 ^cm/s, whereas with sheep CPEC an apparent permeability coefficient of [^14^C]sucrose of approximately 0.8 × 10^-5 ^cm/sec has been measured [39], indicating a tighter monolayer. With rat CPEC monolayers a similar permeability coefficient of sucrose of 0.7 × 10^-5 ^cm/sec was determined at day 8 [12].

The marker enzymes AP and γ-GT showed a decrease in activity over culture time. However, enzymatic activity is often reduced with *in vitro *preparations when compared to tissue *in vivo*, as culture conditions and surrounding environments differ. For example, reduced AP and γ-GT activity was observed in primary brain capillary endothelial cell (BCEC) cultures, with activity decreased during cell proliferation and continuing low even after cells had grown into a confluent monolayer and become fully differentiated [40].

The most interesting aspect in the evaluation of the model was the reduced function of the Mdr1-gene product, p-glycoprotein. Recently, ABC-transporters and in particular p-glycoprotein came in focus as one of the major defense mechanisms of the blood brain barrier protecting the CNS from xenobiotics and maintaining the well balanced homeostasis inside the brain [41-44]. Pgp is also expressed in CP epithelial cells [35,45]. A lack of Pgp function would be in contrast to other organs such as liver, kidney, small intestine or blood brain barrier, where Pgp is documented to play a major role in efflux of compounds and/or transport into the blood stream. However, in this cell culture model, no directed transport of the Pgp substrates, verapamil and rhodamine 123, was observed, which is different from the permeability across capillary endothelial cell monolayers.

There is no data comparing CPEC ABC-transporter expression *in vivo *and *in vitro*. With regard to the BBB, there is one comparative study that investigated expression levels in fresh brain and cultured BCEC [16]. Analysis of Pgp and Mrp1 in whole-brain tissue, isolated brain capillaries and cultured cells showed that gene expression levels varied up to 7-fold after capillary isolation and that expression in cultured cells may be reduced up to 5-fold. The study presented here is the first to provide data on Pgp and Mrp1 mRNA expression in porcine CPEC, and first to present comparative results for Mrp1 mRNA expression in freshly isolated and cultured CPEC on a semi-quantitative basis.

The downregulation of Pgp expression may depend on the culture conditions. There are several alternative explanations for a lack of Pgp functionality [33]. Besides Pgp expression levels being too low to affect CP epithelial drug secretion, the protein is not inserted into the membranes of cultured CPEC at sufficiently high rates. Conversely, Pgp might cluster in vesicles, which are not transported and/or exocytosis does not function effectively in cultured CPEC.

Our finding of a subapical or intracellular localization is in accordance with the observation of previous investigators [35], who detected Pgp in 9- to 12-day-old choroid plexus epithelial cells cultures from rat by indirect immunofluorescence with a punctate or granular staining pattern throughout the cytoplasm. A plasma membrane expression pattern was only occasionally, but not uniformly, observed. Nevertheless, in that study functional activity of Pgp could be demonstrated using paclitaxel and Tc-sestamibi as substrates.

*In vivo *data concerning the functional role of Pgp at the BCSFB are ambivalent: A recent trial with patients suffering from brain tumors showed that there was a trend towards lower paclitaxel concentrations in CSF when given together with tamoxifen [46]. The authors concluded, that agents, which inhibit Pgp, such as tamoxifen, may decrease CSF concentrations of Pgp substrates. However, from another study with intact choroid plexus tissue from guinea pig showing that uptake of the Pgp substrate ritonavir was not influenced by vinblastine, verapamil or other HIV-protease inhibitors, it was concluded that Pgp does not influence the disposition of that drug [47]. Therefore, the functional role of Pgp in intact choroid plexus tissue remains to be clarified.

In contrast, Mrp1 was functionally active in cultured CP cells. Comparative experiments using the model compound FL-MTX, which is transported via a complex interplay of several transport proteins, revealed that these complex processes are fully functional in cultured cells. Results obtained *in vitro *mimicked the distribution observed *in vivo *[37,48]. This finding is supported by studies in mice, showing an increased accumulation of the Mrp1 substrate etoposide in CSF of animals lacking Mrp1 [49]. This indicates, that in CP epithelium Mrp1 may act as a barrier for certain drugs coming from the blood.

## Conclusion

We have established a model of primary choroid plexus epithelial cells from pig brain, which allows the investigation of transport processes. It can be used as a reliable tool for analysis of xenobiotic transport across the blood-cerebrospinal fluid barrier. Expression of Pgp is down-regulated in this model, whereas Mrp1 maintained expression levels and functional activity. The predominantly intracellular localization of Pgp parallels the findings in intact tissue and raises the question about the functional role of this protein in choroid plexus

## Abbreviations

AP, alkaline phosphatase; BCEC, brain capillary endothelial cells; BCSFB, blood-CSF barrier; CNS, central nervous system, CP, choroid plexus; CSF, cerebrospinal fluid; CPEC, choroid plexus epithelial cells; DIC, days in culture; γ-GT, gamma-Glutamyl-Transpeptidase; KRB, Krebs-Ringer buffer; TEER, transepithelial electrical resistance; TTR, transthyretin;

## Competing interests

The author(s) declare that they have no competing interests.

## Authors' contributions

CB carried out the cell isolation, PCR-studies, enzymatic characterisation, Western Blot experiments and fluid secretion studies. VR performed the functional studies with ABC transport proteins and permeability marker analyses. GF conceived of the study, and participated in its design and coordination. All authors read and approved the final manuscript.
